# Early Antibiotic Exposure Alters Intestinal Development and Increases Susceptibility to Necrotizing Enterocolitis: A Mechanistic Study

**DOI:** 10.3390/microorganisms10030519

**Published:** 2022-02-27

**Authors:** Hala Chaaban, Maulin M. Patel, Kathryn Burge, Jeffrey V. Eckert, Cristina Lupu, Ravi S. Keshari, Robert Silasi, Girija Regmi, MaJoi Trammell, David Dyer, Steven J. McElroy, Florea Lupu

**Affiliations:** 1 Department of Pediatrics, Division of Neonatology, University of Oklahoma Health Sciences Center, Oklahoma City, OK 73104, USA; kathryn-burge@ouhsc.edu (K.B.); jeffrey-eckert@ouhsc.edu (J.V.E.); 2 Cardiovascular Biology Research Program, Oklahoma Medical Research Foundation, Oklahoma City, OK 73104, USA; maulin-patel@ouhsc.edu (M.M.P.); cristina-lupu@omrf.org (C.L.); ravi-keshari@omrf.org (R.S.K.); robert-silasi@omrf.org (R.S.); girija-regmi@omrf.org (G.R.); florea-lupu@omrf.org (F.L.); 3 Department of Microbiology and Immunology, University of Oklahoma Health Sciences Center, Oklahoma City, OK 73014, USA; majoi-trammell@ouhsc.edu (M.T.); david-dyer@ouhsc.edu (D.D.); 4 Department of Pediatrics, UC Davis Health, Sacramento, CA 95817, USA; sjmcelroy@ucdavis.edu

**Keywords:** necrotizing enterocolitis, intestinal permeability, intestinal development, antibiotics, preterm infant, microbiome

## Abstract

Increasing evidence suggests that prolonged antibiotic therapy in preterm infants is associated with increased mortality and morbidities, such as necrotizing enterocolitis (NEC), a devastating gastrointestinal pathology characterized by intestinal inflammation and necrosis. While a clinical correlation exists between antibiotic use and the development of NEC, the potential causality of antibiotics in NEC development has not yet been demonstrated. Here, we tested the effects of systemic standard-of-care antibiotic therapy for ten days on intestinal development in neonatal mice. Systemic antibiotic treatment impaired the intestinal development by reducing intestinal cell proliferation, villi height, crypt depth, and goblet and Paneth cell numbers. Oral bacterial challenge in pups who received antibiotics resulted in NEC-like intestinal injury in more than half the pups, likely due to a reduction in mucous-producing cells affecting microbial–epithelial interactions. These data support a novel mechanism that could explain why preterm infants exposed to prolonged antibiotics after birth have a higher incidence of NEC and other gastrointestinal disorders.

## 1. Introduction

Mounting evidence demonstrates the critical role of the gut microbiota on intestinal development and maturation early after birth [1,2]. Perturbation of the gut microbiota during this critical window has been linked to short- and long-lasting effects, including, among others, inflammatory, metabolic, neurologic, and cardiovascular pathologies [3,4,5,6,7,8,9,10,11]. Healthy postnatal microbial colonization is especially critical for preterm infants who are born with underdeveloped intestinal barrier function [12,13]. Unfortunately, microbial colonization is frequently disrupted in this patient population by a multitude of factors, such as the mode of delivery, the types of enteral feeds, and medications, particularly antibiotics [14,15]. While antibiotics are often lifesaving and required for proven infection, overuse of antibiotics is associated with significant neonatal mortality and morbidities, such as sepsis and necrotizing enterocolitis (NEC) [6,16,17,18].

NEC is a gastrointestinal disease characterized by inflammation and necrosis of the small intestine, often leading to rapid bowel perforation, multiorgan failure, and death [19,20]. Approximately 9000 infants develop NEC annually in the United States, 20–40% of whom do not survive. The pathogenesis of NEC remains unclear; however, immature intestinal defenses, abnormal or delayed bacterial colonization of the gut, and formula feeding are considered major risk factors for the development of this devastating disease [21,22]. While short term use of antibiotics is associated with lower risk of NEC [23], prolonged antibiotic treatment in preterm infants during the first two weeks of life is associated with a more than two-fold increased risk of developing NEC [17,24,25]. While a clinical correlation exists between antibiotic use and the development of NEC, the potential causality of antibiotics in NEC development has not yet been demonstrated.

Here, we sought to determine the effects of systemic ampicillin and gentamicin, the most commonly prescribed antibiotic combination among preterm infants [26,27], on intestinal development and susceptibility to an oral bacterial challenge in newborn mice. We hypothesized that early antibiotic treatment (ATB) would negatively alter intestinal development, including the microbiome, and predispose pups to bacteria-induced intestinal injury.

## 2. Materials and Methods

### 2.1. Mouse Experiments

All animal studies were approved by the Institutional Animal Care and Use Committee at the University of Oklahoma Health Sciences Center, OK, USA (IACUC number: 19-062-EFCHI). Mouse pups born to timed pregnant CD-1 dams (Jackson Labs, Bar Harbor, ME, USA) were housed under standard conditions. On P1, pups from both sexes were randomized into two groups: ATB-treated pups and litter mate sham controls. The former group received intraperitoneal (i.p.) injections of ampicillin (100 mg/kg) and gentamicin (7 mg/kg) for 10 days (*n* = 10/group). Body weights were recorded daily in both groups, and pups were returned to dams following daily injections.

### 2.2. Oral Bacterial Challenge

To determine the effect of ATB therapy on an oral bacterial challenge, 1 × 10^7^ colony-forming units (CFU)/g bodyweight *Klebsiella pneumoniae* (ATCC #10031, Manassas, VA, USA)*,* or vehicle control, was administered orally in all pups at P14. The bacterial challenge at P14 was purposely selected to include an ATB-washout period of 4 days, ensuring no residual antibiotics remained at the initiation of bacterial challenge. Pups were euthanized via isofluorane and cardiac puncture 10 h post-bacterial gavage, and blood and tissues were collected. This procedure resulted in four treatment groups: (i) Control (*n* = 10); (ii) pups exposed to ATB for 10 days followed by vehicle at P14 (ATB, *n* = 15); (iii) pups not treated with ATB but exposed to bacteria at P14 (Bac, *n* = 17); and (iv) pups treated with ATB for 10 days followed by bacteria at P14 (ATB + Bac, *n* = 22).

### 2.3. Tissue and Blood Collection

Post-euthanasia, the small intestine was resected and its length was measured. Sections from the terminal ileum were fixed in 10% buffered formalin for histology and immunohistochemical staining or Carnoy’s fixative (60% ethanol, 30% chloroform, 10% glacial acetic acid), as appropriate.

### 2.4. Quantification of Crypt Depth, Villus Height, and Paneth and Goblet Cell Numbers

Paraffin-embedded sections (5-μm thick) from the terminal ileum were cut, deparaffinized, rehydrated, and stained with hematoxylin and eosin (H&E) for morphological analysis. Villus heights and crypt depths were measured using NIH ImageJ software from at least 50 well-oriented villi and crypts in three animals per group. For determination of Paneth and goblet cell numbers, sections were stained with Periodic Acid Schiff (PAS)/Alcian blue (AB) (MilliporeSigma, St. Louis, MO, USA), as previously described [28].

### 2.5. Histological Scoring

The severity of intestinal injury secondary to the bacterial challenge was assessed using the previously published NEC-like intestinal injury scoring system [22,29]. Histological changes were scored by a blinded evaluator and graded as follows: Grade 0 (no damage); Grade 1 (mild; slight submucosal and/or lamina propria separation); Grade 2 (moderate; moderate separation of the submucosa and/or lamina propria); Grade 3 (severe; severe separation of the submucosa and/or lamina propria or severe edema in the submucosa and regional villous sloughing); and Grade 4 (transmural necrosis). Scores were based on the highest score observed on three to five sections in a specimen. Only animals with a score of 2 or above were considered to have developed NEC-like intestinal injury [29].

### 2.6. Immunofluorescence

Paraffin-embedded sections (5-μm thick) were deparaffinized, rehydrated, then steamed for 30 min in citrate buffer for antigen retrieval. Sections were blocked with 5% goat serum in Tris-buffered saline with 0.05% Tween 20, incubated with a primary antibody for Ki-67 (1:100, ThermoFisher Scientific, Waltham, MA, USA) overnight, followed by Alexa Fluor 647-conjugated secondary antibody. For detection of apoptosis, ileal sections were stained with terminal deoxynucleotidyl transferase dUTP nick end labeling (TUNEL) using the In-Situ Cell Death Detection Kit, TMR red (Roche Diagnostics, Indianapolis, IN, USA), as per the manufacturer’s instructions. All sections were mounted with VECTASHIELD Antifade Mounting Medium (Vector Laboratories, Burlingame, CA, USA) containing DAPI (4′,6-diamidino-2-phenylindole) as a nuclear stain. Slides were visualized with the C1 laser scanning Confocal Microscope System (Nikon, Melville, NY, USA).

### 2.7. Fluorescence In Situ Hybridization (FISH) for Bacteria and Co-Staining with Lectin

Dual staining for FISH and lectin was carried out as previously described [30,31]. Briefly, deparaffinized sections were incubated at 37 °C overnight with Alexa Fluor 488-conjugated gamma-Proteobacteria Phylum GAM42a (5′-GCC TTC CCA CAT CGT TT-3′), which recognizes bacteria belonging to the γ-Proteobacteria class, in hybridization buffer (20 mM Tris-HCl, pH 7.4, 0.9 M NaCl, 0.1% sodium dodecyl sulfate). Sections were rinsed with wash buffer (20 mM Tris-HCl, pH 7.4, 0.9 M NaCl) and PBS twice each, and were co-labeled with fluorochrome-conjugated lectin (Ulex europaeus agglutinin I, UEA1, DyLight^®^ 649, DL-1068, Vector Laboratories) then mounted with VECTASHIELD. The fluorescence intensity of lectin was quantified using ImageJ software and represented as the integrated fluorescence intensity of the lectin relative to total DAPI values per tissue section. Slides were stained under the same conditions and at the same time.

### 2.8. Intestinal Permeability Assay

Gut barrier integrity was assessed using fluorescein isothiocyanate (FITC)-labeled dextran (molecular weight 4000) (Sigma-Aldrich, St. Louis, MO, USA), as previously described [32]. In brief, four hours before euthanasia, pups were administered FITC-dextran at a dose of 44 mg/100 g body weight. After collection, blood FITC-dextran concentration was measured by spectrophotofluorometry (Tecan, Maennedorf, Switzerland) at an excitation wavelength of 480 nm and an emission wavelength of 520 nm, after standard concentration curves were established.

### 2.9. Measurement of Tissues Cytokines

Proinflammatory IL (interleukin)-1β, tumor necrosis factor (TNF)-α, interferon (IFN)-γ, growth-regulated oncogene (GRO)-α, and anti-inflammatory IL-10 were measured in plasma and intestine using ProcartaPlex Mouse Cytokine Panel (eBioscience, San Diego, CA, USA) based on Luminex technology, as per the manufacturer’s instructions. Small intestinal samples were homogenized using a bead beater (Next Advance, Troy, NY, USA) in buffer containing phosphatase and protease inhibitors (#’s 524625 and 535140, respectively; Millipore, Burlington, MA, USA) and PMSF (Sigma-Aldrich). Samples were run on a BioPlex 200 (Bio-Rad, Hercules, CA, USA). Final cytokine levels were normalized to total protein concentration (mg/mL) and reported as picogram/mL for plasma and picogram/mg tissue.

### 2.10. Cecal Microbial Composition

Following euthanasia, cecal contents from ATB and control mouse pups were collected in bead tubes using the Stool DNA Isolation Kit (Norgen Biotek, Thorold, ON, Canada). Bacterial DNA extraction was performed according to the manufacturer’s instructions. DNA quantity and quality were characterized using the NanoDrop Lite Spectrophotometer (ThermoFisher Scientific). PCR amplification of the hypervariable V3–V4 segment of the 16S rRNA gene was achieved using an Illumina kit, as previously described [28]. Libraries were sequenced on the Illumina MiSeq platform using the MiSeq Reagent Kit v3 for 600 cycles to collect 300 bp paired-end reads. The generated data (up to 18 Gb) were filtered for quality and clustered into Amplicon Sequence Variants (ASVs) using DADA2 [33]. The ASVs were subsequently classified against the Greengenes 16S rRNA database [34].

### 2.11. Statistical Analysis

Statistical analysis was conducted using GraphPad Prism version 6.0 (GraphPad Software, San Diego, CA, USA). Results are depicted as mean ± standard error of the mean (SEM). Differences between the two groups were analyzed by ratio-paired *t*-tests and student’s *t*-tests. Multiple groups were analyzed by repeated measures using one-way or two-way analysis of variance (ANOVA) with post hoc Tukey, Mann–Whitney U, or Kruskal–Wallis tests, as appropriate. Differences between or among groups were considered significant at *p *< 0.05. Microbiome statistics were performed using the following: To determine the β-diversity distances within and between groups, PERMANOVA was used to compare Bray–Curtis distances [35]. The Kruskal–Wallis test was used to determine the statistical significance of α-diversity. Linear discriminant analysis [36] was used to determine the differential taxonomic abundance and PICRUSt2-derived [37] pathway data. Finally, *p*-values were adjusted using the false discovery rate (FDR).

## 3. Results

### 3.1. Antibiotic Treatment Negatively Impacts Postnatal Intestinal Development

**Figure 1:**
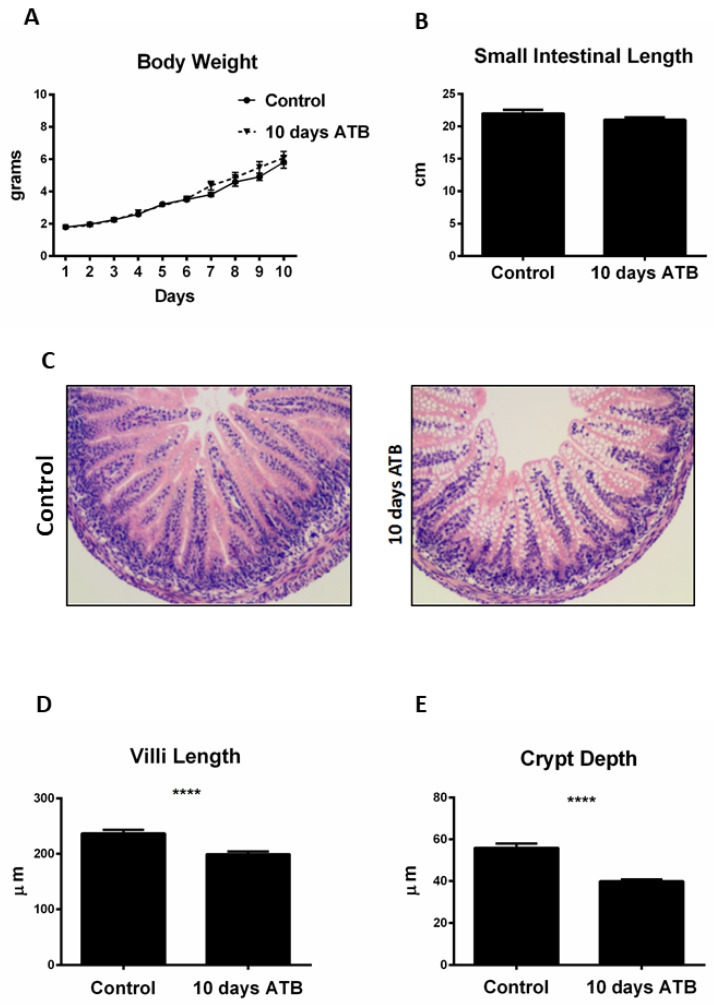
ATB treatment negatively impacts villi length and crypt depth. (**A**) Effect of ATB for 10 days on mouse pup body weights and (**B**) small intestinal lengths. (**C**) Representative hematoxylin and eosin ileal sections from both groups. ATB treatment is associated with shorter villi length (**D**) and crypt depth (**E**) measured in µm (*n* > 50 crypts and villi; **** *p* < 0.0001). Data presented as mean ± SEM. ATB: antibiotic treatment.

Previous studies have shown that early oral antibiotic therapy in mouse pups is associated with negative effects on intestinal development and immunity [38,39]. The effects of systemic, standard-of-care antibiotics, specifically ampicillin and gentamycin, on intestinal development have not previously been addressed. We sought to determine the effects of translationally relevant ATB on overall weight gain and small intestinal length and microarchitecture (villi length and crypt depth). Pups from both groups were weighed daily from birth until the end of treatment (P10). No significant differences were observed in daily weight gain (Figure 1A), with control pups gaining an average of 0.49 g/day and ATB pups gaining 0.52 g/day. ATB treatment had no effect on the small intestine length (Figure 1B) compared to controls (ATB: 22 ± 1.7 cm; Control: 21.02 ± 0.84 cm; *p* = 0.48). However, pups in the ATB group displayed significantly shorter villi lengths (ATB: 199.4 ± 4.6 µm; Control: 236.6 ± 6.8 µm; *p* < 0.0001) and crypt depth (ATB: 38.89 ± 0.92 µm; Control: 55.9 ± 0.21 µm; *p* < 0.0001) compared to controls (Figure 1C–E).

**Figure 2:**
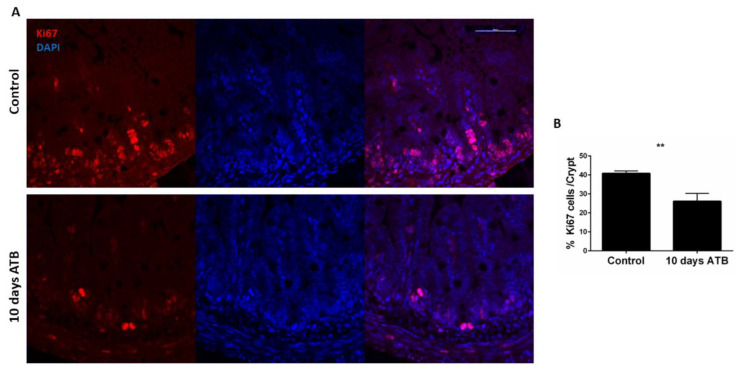
Effect of systemic ATB on intestinal epithelial proliferation. (**A**) Immunostaining of ileal sections from pups in control and ATB groups with antibody against Ki67 (red), and DAPI (blue). Magnification × 100, scale bar 100 µm. (**B**) Quantification (*n* > 50 crypts; ** p = 0.002) of Ki67 staining per crypt in both groups. Data presented as mean ± SEM. ATB: antibiotics.

To determine the contribution of intestinal epithelial apoptosis or proliferation on the microarchitecture, we performed TUNEL and Ki67 staining on ileal sections. Pups in the ATB group had significantly fewer Ki67-positive cells per crypt, indicating reduced intestinal epithelial proliferation in the ATB group compared to control (Figure 2A,B; ATB: 26 positive cells/crypt; Control: 40 positive cells/crypt; *p* = 0.002). In contrast, ATB treatment induced no changes in intestinal epithelial apoptosis (data not shown).

### 3.2. ATB Treatment Significantly Decreases Goblet and Paneth Cell Numbers

**Figure 3:**
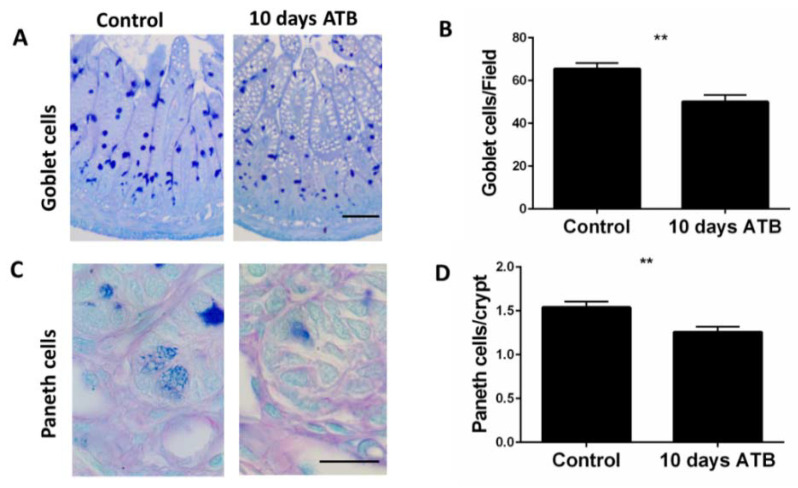
Effect of systemic ATB on goblet cell and Paneth cell numbers. Representative PAS staining and corresponding quantification of numbers of goblet cells per field ((**A**,**B**) ** *p* =0.0014). Magnification: 20×; scale bar: 50 µm. Representative images for Paneth cells, identified by the distinct secretory granules, and corresponding quantification of numbers per crypt ((**C**,**D**) ** *p* = 0.001). Magnification: 40×; scale bar: 20 µm. Data presented as mean ± SEM. ATB: antibiotics.

We next examined the impact of systemic ATB on goblet cells and Paneth cells. Both cells are key components of the mucosal barrier and play important roles in host defense against intestinal pathogens. Compared to controls, ATB pups had a significantly lower number of PAS-positive goblet cells per field (Figure 3A,B; Control: 65.5 cells/field; ATB: 50 cells/field; *p* = 0.0014). Similarly, Paneth cells, identified by the distinct secretory granules at the base of the crypts, were slightly but significantly lower in number in the ATB groups compared to controls (Figure 3C,D; ATB: 1.25 cells/crypt; Control: 1.54 cells/crypt; *p* = 0.0017).

### 3.3. Antibiotic Treatment Alters Cecal Bacterial Diversity and Composition

**Figure 4:**
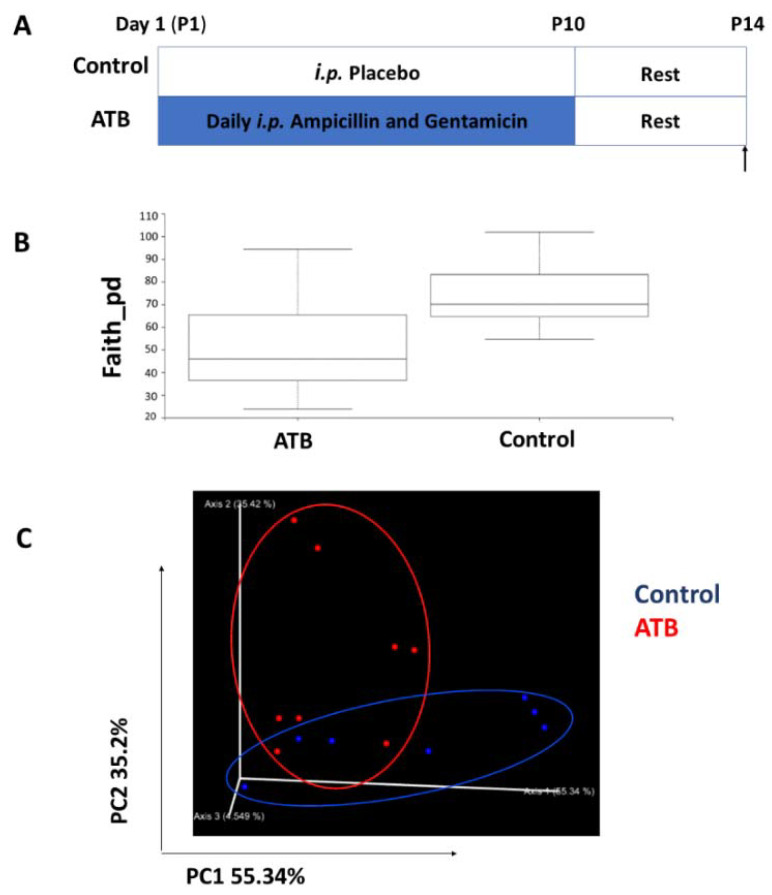

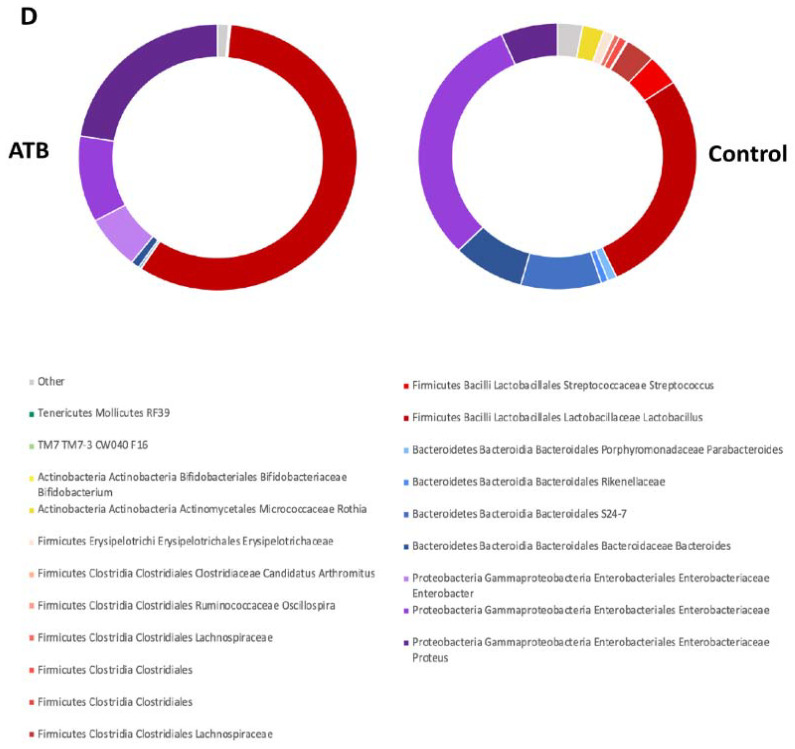
Analysis of cecal microbiota composition between groups. (**A**) Mouse pups were given ATB i.p. once daily for 10 days, and cecal contents were collected at P14, after a 4-day recovery. (**B**) Faith’s phylogenetic diversity metric displays species richness of mouse pups differentiated by treatment (Kruskal–Wallis *p* = 0.0491). (**C**) PCoA plot displaying Bray–Curtis beta diversity matrix (Control = blue; ATB = red). Percent confidence values for each distance matrix are displayed on the axes in two dimensions. PERMANOVA results: F = 3.10; *p* = 0.037. (**D**) Taxonomy plots were generated using a Naïve Bayes classifier trained on the most recent Greengenes 16S rRNA database. ASV reads were taxonomically classified and filtered for genera > 0.5% of total microbiome composition for any one sample. Genera falling below the 0.5% mark were placed in “other” (grey). ATB: antibiotics; PCoA: principal components analysis.

To determine the effect of systemic ATB therapy on the intestinal microbiome, cecal contents from ATB and control mouse pups were collected at P14, following a 4-day washout period (Figure 4A). As expected, pups in the ATB group had significantly lower alpha diversity relative to controls, as assessed by Faith’s phylogenetic diversity (Figure 4B, *p* = 0.0491). Furthermore, unweighted UniFrac principal coordinates analysis (PCoA) indicated an impact of ATB on the beta diversity of the gut microbiota (Figure 4C, PERMANOVA weighted Unifrac distance F = 3.486, *p* = 0.037). Taxonomy plots of cecal microbiota from both groups are displayed in Figure 4D. Overall, Firmicutes and Bacteroidetes bacteria dominated both groups. However, ATB pups showed an increased abundance of Bacteroidales and Micrococaccaceae relative to controls.

**Figure 5:**
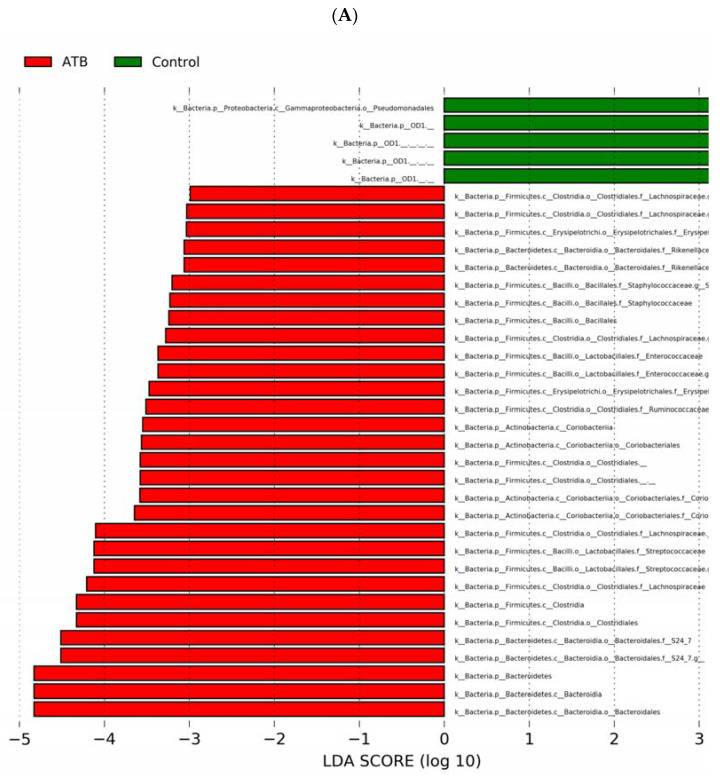

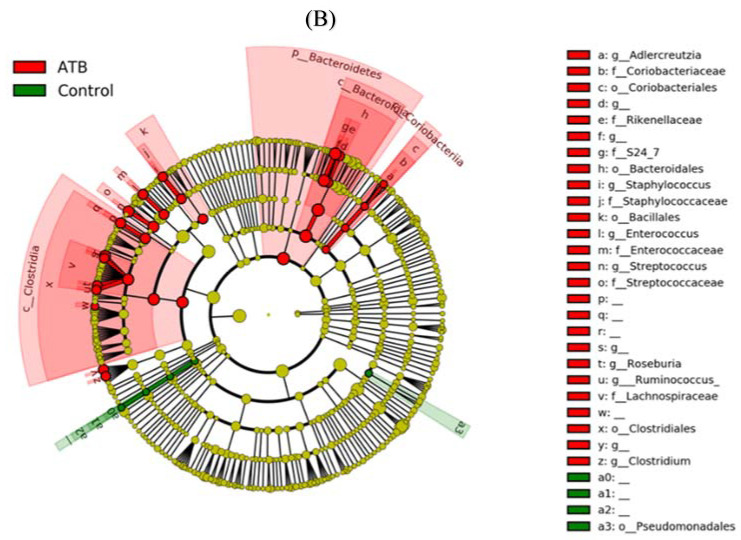
LEfSe analysis of cecal microbiome from mouse pups in control and ATB groups. (**A**) LDA scores of significantly different bacteria between control (green) and ATB-treated (red) pups. (**B**) Cladogram utilizing LEfSe indicates the phylogenetic distribution of fecal microbes associated with the control (green) and ATB-treated (red) pups. ATB: antibiotics; LEfSe: linear discriminant analysis effect size; LDA: linear discriminant analysis.

Next, we performed LEfSe analysis to further characterize differences between the groups. The relative abundance of bacteria in the phyla Bacteroidetes, Firmicutes, and Actinobacteria was increased, while Proteobacteria was slightly decreased in cecal contents of ATB pups compared to controls. At the genus level, Streptococcus, Adlercreutzia, Staphylococcus, Enterococcus, Ruminococcus, and Clostridium exhibited increased relative abundances in the ATB group compared to controls (Figure 5A,B; linear discriminant analysis (LDA) score (log 10) > 3).

### 3.4. Systemic ATB Increases Intestinal Permeability and Inflammatory Tone

**Figure 6:**
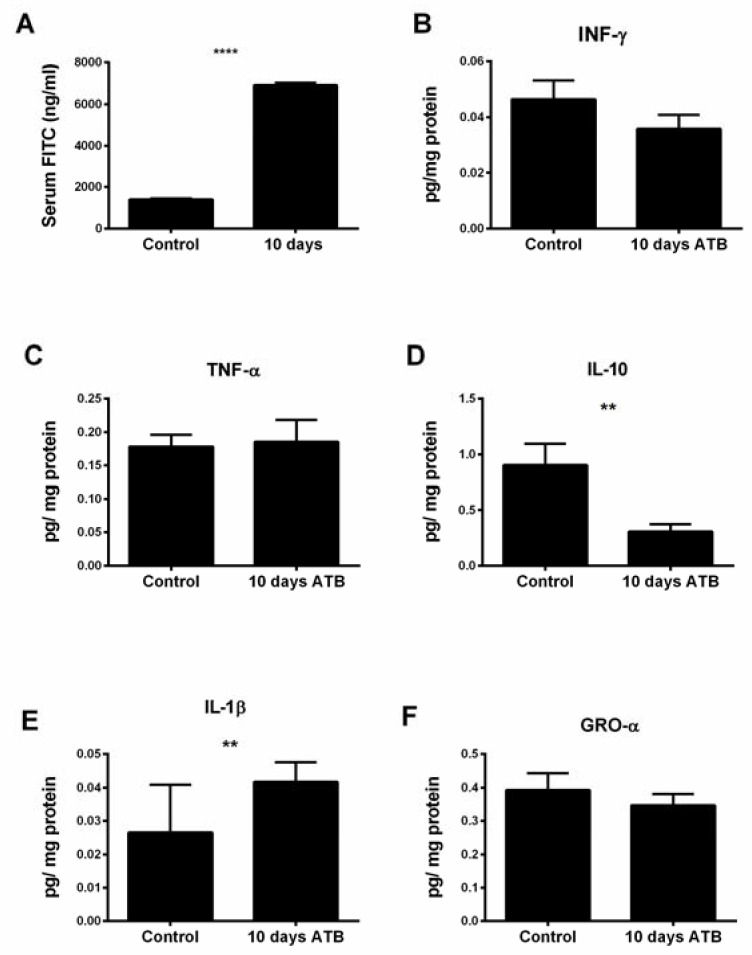
Effect of systemic antibiotics on intestinal permeability and ileal cytokine expression. (**A**) Serum FITC-dextran concentrations in pups in control and ATB groups; **** *p* < 0.0001. (**B**–**F**) Ileal cytokine expression from pups in control and ATB groups; ** *p* < 0.01. Values denote mean ± SEM (*n* ≥ 6). ATB: antibiotics; FITC: fluorescein isothiocyanate; IFN: interferon; IL: interleukin; GRO: growth-regulated oncogene.

Next, we investigated the impact of the above ATB-induced changes on intestinal permeability and inflammation. Notably, pups in the ATB group had a nearly 4-fold elevation in serum levels of FITC-dextran compared to controls, suggesting a significantly weakened intestinal barrier (Figure 6A; *p* < 0.0001). To gain further insight into the effect of ATB on inflammatory cytokines in the intestine, concentrations of IFN-γ, TNF, IL-10, IL-1β, and GRO- α were measured. No differences were seen in the ileal levels of IFN-γ, TNF-α, and GRO-α. However, a significant increase was seen for IL-1β (*p* = 0.007) and a decrease in the anti-inflammatory IL-10 (*p* = 0.003) was observed in the ATB pups compared to controls (Figure 6B–F).

### 3.5. ATB Increases Susceptibility to Subsequent Klebsiella-Induced Intestinal Injury

**Figure 7:**
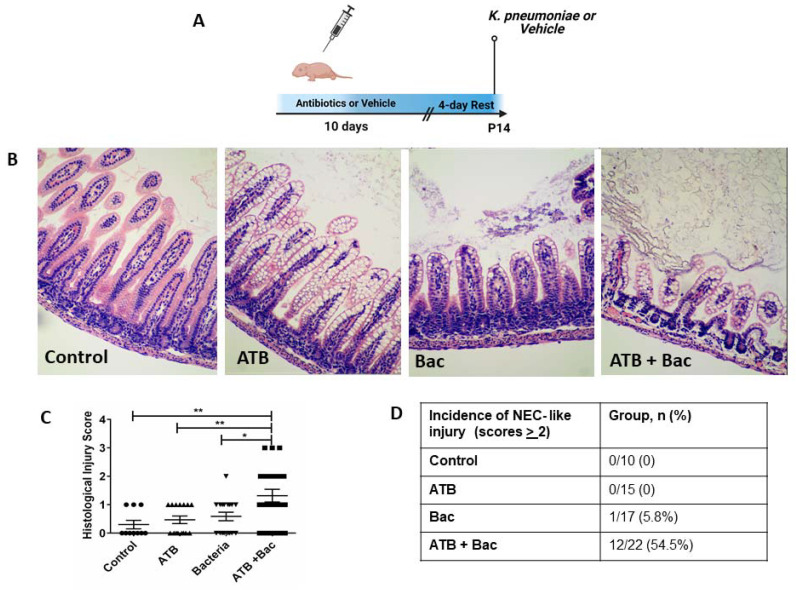
ATB treatment for 10 days increases susceptibility to bacteria-induced intestinal injury. (**A**) Experimental design. (**B**) Representative hematoxylin and eosin ileal sections from control, ATB, Bac, and AT + Bac groups. Magnification: 20×. (**C**) NEC-like histological injury scoring; ** *p* < 0.01, * *p* < 0.05. (**D**) Incidence of NEC-like intestinal injury score >2 among groups. Scores were determined by a blinded investigator. Values denote mean ± SEM. ATB: antibiotics; Bac: bacteria.

While our results showed that systemic ATB treatment negatively affects intestinal development and increases gut permeability, the impact of these changes on overall gut protection against a bacterial challenge was still unclear. Therefore, we investigated the potential susceptibility of mouse pups to an oral dose of *K. pneumoniae*, a bacterium known to play a significant role in animal and human intestinal inflammatory injury and NEC [29,40,41,42]. As shown in Figure 7A, pups were randomized after birth to either 10 days of i.p. ATB or vehicle control. After a 4-day recovery, pups were further randomized to receive either oral *K. pneumoniae* or vehicle at P14, creating a total of four experimental groups (Supplementary Figure S1): Control (10-day vehicle, vehicle at P14), ATB (10-day antibiotics, vehicle at P14), Bac (10-day vehicle, *K. pneumoniae* at P14), and ATB + Bac (10-day antibiotics, *K. pneumoniae* at P14). Following oral *K. pneumoniae* or vehicle, pups were returned to dams and monitored for ten hours, then euthanized for blood and tissue collection. Histological injury scoring by a blinded observer revealed a significantly greater overall score in the ATB + Bac group (Figure 7B,C; 1.32 ± 0.29) compared to ATB (0.46 ± 0.13; *p* = 0.007), Bac (0.59 ± 0.15; *p* = 0.02), or control (0.3 ± 0.29; *p =* 0.004). Histopathological analysis indicated significant damage to intestinal tissue architecture in 54% of mouse pups in the ATB + Bac group, with obvious vacuolation, mucosal edema, loss of villous integrity, intramural air, and some areas of villous sloughing (Figure 7D). Except for one pup in the Bac group, no pups in other groups had an intestinal injury, indicating the pups are generally protected from an oral bacterial challenge if not made susceptible through antibiotic treatment.

**Figure 8:**
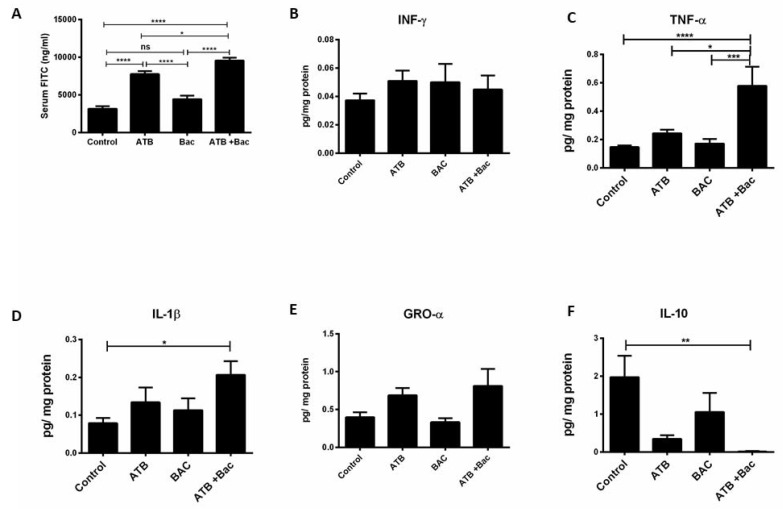
Effect of systemic antibiotics on intestinal permeability and ileal cytokine expression. (**A**) Serum FITC concentration in control, ATB, Bac, and AT + Bac pups; * *p* < 0.05, **** *p* < 0.0001. (**B**–**F**) Ileal cytokine expression from pups in the four groups; * *p* < 0.05, ** *p* < 0.01, *** *p* < 0.001, **** *p* < 0.0001. Values denote mean ± SEM (n ≥ 10). ATB: antibiotics; Bac: bacteria; IFN: interferon; IL: interleukin; GRO: growth-regulated oncogene.

In support of the above findings, pups exposed to the combination of ATB + Bac exhibited a significantly increased intestinal permeability, as assessed by serum FITC levels, compared to pups in the control, Bac, and ATB groups (Figure 8A; *** p < 0.0001, * *p* < 0.05, *** *p* < 0.0001, respectively). Furthermore, ileal expression of TNF and IL-1β were significantly increased, while IL-10 levels were decreased in the ATB + Bac group compared to the control, ATB, and Bac pups (Figure 8C–F). While ATB treatment alone was associated with increased intestinal permeability, no significant differences were seen in ileal cytokine levels compared to controls. More importantly, oral *K. pneumoniae* alone in the Bac group did not induce intestinal injury, inflammation, or increased intestinal permeability as indicated by similar expression of ileal cytokine levels, serum FITC levels, and ileal histology (Figure 8B–F).

**Figure 9:**
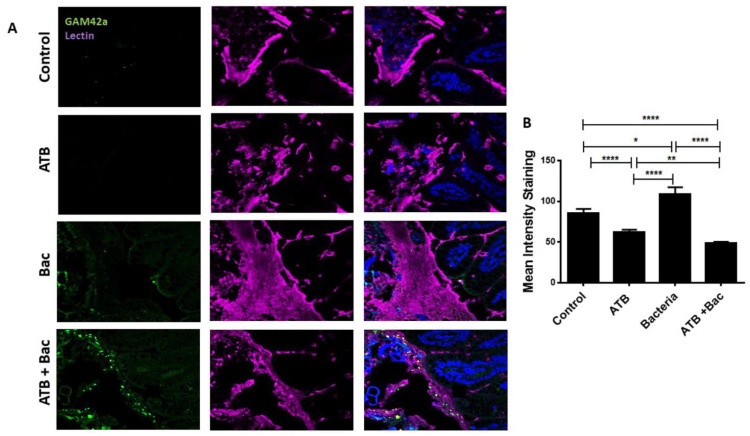
ATB treatment is associated with a thinning of the mucus layer and enhanced bacterial–epithelial interaction. (**A**) Representative images show dual staining for UEA1 lectin (purple) and bacteria (FITC, green). Blue, DAPI for nuclear staining. (**B**) Mean fluorescence intensity for lectin relative to DAPI was measured for each group using ImageJ software. * *p* < 0.05, ** *p* < 0.01, **** *p* < 0.0001. Values denote mean ± SEM. ATB: antibiotics; Bac: bacteria; UEA1: ulex europaeus agglutinin 1; FITC: fluorescein isothiocyanate; DAPI: 4′,6-diamidino-2-phenylindole; SEM: standard error of the mean.

To elucidate the mechanism for the increased susceptibility of ATB-treated pups to oral *K. pneumoniae*, we interrogated the potential roles of goblet cells and the mucus layer. Goblet cells cover the surface of enterocytes with a barrier of mucus, protecting the intestine against invading pathogens [43]. We hypothesized that an ATB-induced reduction in goblet cells could lead to altered mucus production in the intestine, allowing direct interaction between bacteria and the host epithelium. In line with our hypothesis, ATB treatment resulted in a reduced goblet cell number compared to controls, indicated by the reduction in Muc2 positive cells per field (Supplementary Figure S2). Next, we performed dual staining for lectin, a component of mucus, and luminal bacteria via fluorescence in situ hybridization (FISH) with the GAM42a bacterial probe, identifying bacteria of the γ-Proteobacteria class. In the ATB-treated pups, GAM42a signal was not detected, and the mucus layer, while present, was fragmented, with a lower staining intensity compared to controls (Figure 9A,B; *p* < 0.0001). Notably, pups in the Bac group had significantly increased mucus barriers and visible separation of the epithelium from the GAM42a signal in the lumen. In contrast, pups in the ATB + Bac group had increased GAM42a signals in the lumen and visibly thinner mucus layers, thus potentially allowing for contact between bacteria and the intestinal epithelium.

## 4. Discussion

NEC is the most common gastrointestinal emergency in the neonatal intensive care unit (NICU) [44,45], with a high economic burden accounting for $1B/year in the U.S. alone [46]. Although the pathogenesis of NEC is unclear, studies suggest NEC is a result of complex interactions among feeding, abnormal bacterial colonization, exaggerated inflammatory response, and an immature intestinal epithelium [47]. Recent studies highlight the association between prolonged antibiotic therapy in preterm infants and the development of NEC [6,16,17,18]. Importantly, approximately 30–50% of preterm infants are exposed to prolonged antibiotic treatment due to their high risk for infection. While a role for antibiotic-induced dysbiosis in NEC has been proposed [27,48], the potential mechanisms involved are not understood. 

In this study, we examined the impact of the commonly used systemic ATB, ampicillin, and gentamicin, in neonatal mouse pups. We showed that systemic ATB treatment for ten days altered the intestinal microbiome and negatively impacted intestinal development and barrier function. ATB-treated pups had significantly reduced villus height and crypt depth compared to controls. Furthermore, ileal sections from ATB pups had decreased proliferating cell numbers as indicated by Ki-67 staining, and lower numbers of Paneth and goblet cells. We also found ATB-treated pups were more susceptible to NEC-like intestinal injury secondary to an oral bacterial challenge, likely due to a reduction in mucous-producing cells affecting microbial-epithelial interactions. These results provide new insights into the potential mechanism underlying increased NEC development among preterm infants treated with antibiotics. 

Several factors should be considered when evaluating the effect of antibiotics on gut microbiota, specifically the spectrum of coverage, route of administration, dose, and duration of treatment [49]. While previous studies demonstrate the drastic effects of enteral antibiotic treatment on the composition and diversity of the intestinal microbiota in animal models [39,42], the effects of systemic ampicillin and gentamicin were unexplored. Not surprisingly, pups treated with this combination displayed reduced species richness, a widely recognized measure of general microbiome health [50,51], compared to controls. Similar to other studies using enteral ampicillin and gentamicin, ATB-treated pups had a decreased abundance of Gammaproteobacteria on day 14 [52] with an increased relative abundance of *Streptococcus*, *Adlercreutzia*, *Staphylococcus*, *Enterococcus*, and *Clostridium* compared to controls. Though Gammaproteobacteria “bloom” has been described in preterm infants who develop NEC and has been implicated in the pathogenesis of NEC, our finding of its increased abundance is likely the result of gentamycin, an aminoglycoside known to exhibit bactericidal effects against this specific class. Further studies are needed to determine the contribution of these findings to our results. In addition, while *Staphylococcus*, *Enterococcus*, and *Clostridium* and their products have been associated with increased permeability and intestinal injury in animal models [53,54], their contribution to increased intestinal permeability and inflammation in our model needs to be further investigated.

Our results expand upon previous reports that antibiotic use leads to alterations in the developmental pattern of goblet and Paneth cells [55]. Both cell types are key components of the mucosal barrier and play important roles in intestinal stem cell homeostasis, development of the microbiome, and host defense against intestinal pathogens. Paneth cells are a major source of antimicrobial compounds, such as lysozymes and defensins [56]. Impairment of the number or function of these cells is associated with a reduction in clearance of bacterial pathogens and the development of inflammatory bowel disease and NEC [29,57,58,59,60]. Goblet cells are specialized intestinal epithelial cells with a well-established role in innate immunity through the secretion of mucins and maintenance of the mucus layer. Muc2, the main structural component of the intestinal mucus barrier, is heavily glycosylated with sugars that bind microbes, preventing the direct interaction of microbes with the host intestinal epithelium. Changes in this protective layer can alter the exposure of the underlying epithelium to pathogens and foreign antigens [43]. Mice deficient in Muc2 production are more susceptible to intestinal infections [61,62] and spontaneously develop colitis [63], suggesting mucus production protects against enteric pathogens. Notably, in both human and rodent models of NEC, the number of Muc2-producing goblet cells is significantly reduced. Muc2 deficient mice develop a more severe NEC pathology compared to those with normal mucin. [64,65,66,67]. Collectively, these data indicate a possible role for goblet cells in the development of NEC.

Previous studies demonstrate the importance of microbial colonization on intestinal epithelial cell differentiation and maturation [68]. This differentiation includes a dramatic increase in goblet cell numbers likely due to exposure to bacteria and their products in the lumen after birth. Compared with conventionally housed mice, germ-free mice have significantly fewer goblet cells and are more susceptible to intestinal invasion and inflammation [2,3,69]. When colonized with commensal bacteria or probiotics, goblet cells quickly increase in both size and number [2]. Importantly, abnormal or delayed colonization of the gut secondary to antibiotics is associated with an increased risk of intestinal infection and injury due to defective intestinal mucus barrier [62], allowing direct interaction between microbes and the intestinal epithelium. Indeed, our ATB-treated pups had a significantly lower number of Muc2-positive cells in the intestinal epithelium compared to controls. In contrast, pups challenged with bacteria alone had a significantly increased mucus intensity staining, suggesting increased goblet cell secretion, with a visible separation of the intestinal epithelium from the bacteria in the lumen. These data are in line with previous studies showing mucin production or release from goblet cells is driven by the presence of commensal bacteria and pathogens [70]. We also found that mice pretreated with ATB suffered NEC-like intestinal injury when subsequently challenged with oral pathogenic bacteria. The histological injury was associated with increased intestinal permeability and inflammatory cytokine expression. Further, dual lectin/bacteria staining revealed a fragmented mucus layer in the small intestine of ATB pups and apparent direct interaction of bacteria with the intestinal epithelium. It is plausible that prolonged ATB exposure in preterm infants after birth potentially prevents the growth of a subset of normal gut microbes, negatively affecting intestinal maturation and differentiation. Alternatively, ATB treatment could lead to an increased predominance of mucin-degrading bacteria in the preterm infant stools. The resultant effect would be a thinning of the protective mucus layer and increased bacterial-epithelial interaction or activation, eliciting excessive pro-inflammatory responses and potentially leading to epithelial necrosis, as in NEC [71,72,73].

Our study is subject to several limitations. First, due to the inherently discontinuous nature of the mucus layer in the small intestine, mucus thickness could not be measured. Instead, we used intensity staining as a surrogate for mucus production and thickness. Second, ATB therapy in mouse pups may contribute to delays in intestinal development through an additional mechanism that was not evaluated in this study, such as direct interactions with the intestinal epithelium. Additionally, the contribution of the effects on stem cell function on those findings was not evaluated. Lastly, though our data demonstrate differences in the intestinal microbial composition due to systemic ampicillin and gentamicin, further investigation is needed to evaluate if negative effects in the small intestine are due to the absence or presence of specific bacteria and their metabolites, rather than alterations in the microbiome collectively. 

## 5. Conclusions

In summary, our novel data indicate that systemic ATB treatment negatively affects intestinal barrier function, epithelial cell proliferation, and enterocyte differentiation to Paneth and goblet cells. Moreover, ATB treatment increased the susceptibility of mouse pups to NEC-like intestinal injury, likely due to the thinner mucus lining and alterations to the microbiome. These findings could provide new insight into the association between antibiotic treatment and the development of NEC. 

## Data Availability

The data presented in this study are available on request from the corresponding author.

## Supplementary Materials

The following supporting information can be downloaded at: https://www.mdpi.com/article/10.3390/microorganisms10030519/s1, Figure S1: Description of the four experimental groups. Figure S2: Goblet cell numbers in the four experimental groups.
