# Supplementary figures and images for: Early Antibiotic Exposure Alters Intestinal Development and Increases Susceptibility to Necrotizing Enterocolitis: A Mechanistic Study
Source: Microorganisms. 2022 Feb 27;10(3):519. doi: 10.3390/microorganisms10030519 (PMC8951210; doi:10.3390/microorganisms10030519)

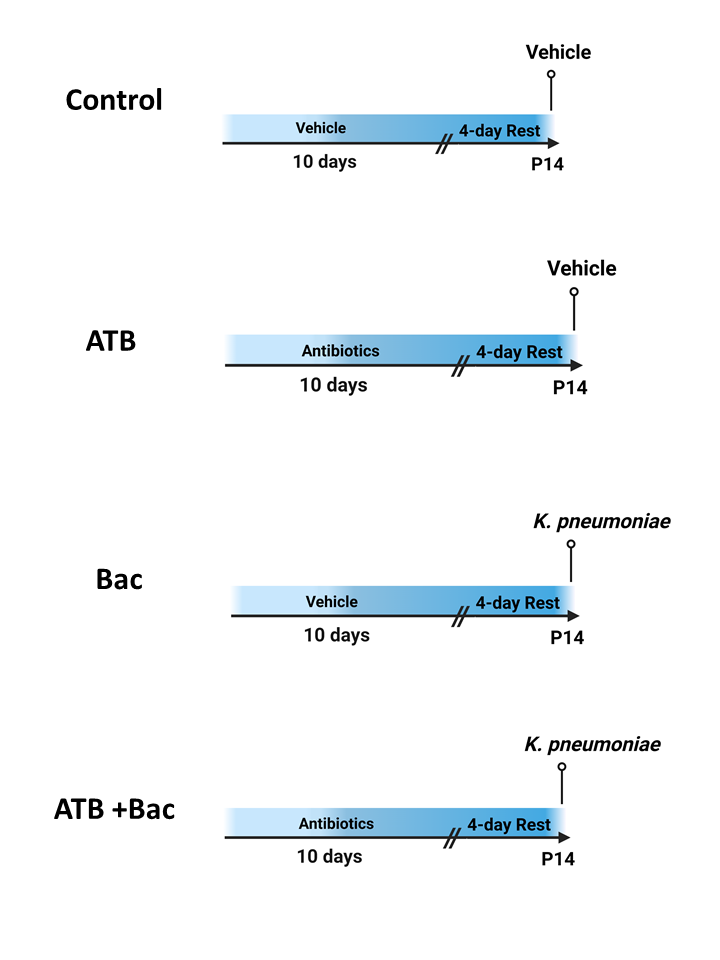

Supplement: Supplementary file 1 [file microorganisms-10-00519-s001.zip › Figure S1. Description of the four experimental groups.tif]

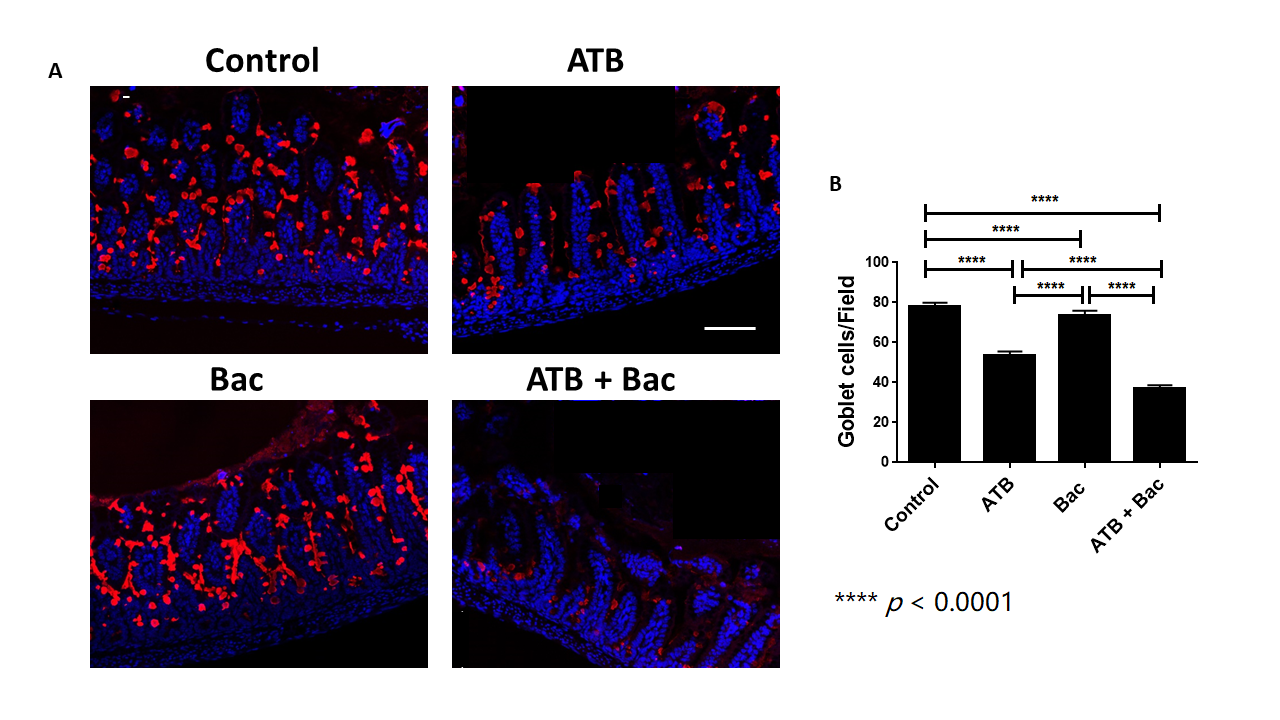

Supplement: Supplementary file 1 [file microorganisms-10-00519-s001.zip › Figure S2. Goblet cell numbers in the four experimental groups.tif]
